# Glycogen synthase kinase-3-mediated phosphorylation of serine 73 targets sterol response element binding protein-1c (SREBP-1c) for proteasomal degradation

**DOI:** 10.1042/BSR20150234

**Published:** 2016-01-15

**Authors:** Qingming Dong, Francesco Giorgianni, Sarka Beranova-Giorgianni, Xiong Deng, Robert N. O'Meally, Dave Bridges, Edwards A. Park, Robert N. Cole, Marshall B. Elam, Rajendra Raghow

**Affiliations:** * Department of Veterans Affairs Medical Center, 1030 Jefferson Avenue, Memphis, TN 38104, U.S.A.; † Department of Pharmacology, College of Medicine, the University of Tennessee Health Science Center, 874 Union Avenue, Memphis, TN 38163, U.S.A.; ‡ Department of Pharmaceutical Sciences, College of Pharmacy, the University of Tennessee Health Science Center, 881 Madison Avenue, Memphis, TN 38163, U.S.A.; § Mass Spectrometry and Proteomics Facility, Johns Hopkins University School of Medicine, Baltimore, Maryland 21205, U.S.A.; ║ Department of Physiology, College of Medicine, the University of Tennessee Health Science Center, 894 Union Avenue, Memphis, TN 38163, U.S.A.; ¶ Children's Foundation Research Institute, Le Bonheur Children's Hospital, Department of Pediatrics, the University of Tennessee Health Science Center, 50 North Dunlap, Memphis, TN 38103, U.S.A.

**Keywords:** cdc4 phosphodegron (CPD), GSK-3, mass spectrometry (MS), phosphorylation, sterol regulatory element binding protein (SREBP), ubiquitination

## Abstract

Sterol regulatory element binding protein-1c (SREBP-1c) is a key transcription factor that regulates genes involved in the *de novo* lipid synthesis and glycolysis pathways. The structure, turnover and transactivation potential of SREBP-1c are regulated by macronutrients and hormones via a cascade of signalling kinases. Using MS, we have identified serine 73 as a novel glycogen synthase kinase-3 (GSK-3) phosphorylation site in the rat SREBP-1c purified from McA-RH7777 hepatoma cells. Our site-specific mutagenesis strategy revealed that the turnover of SREBP-1c, containing wild type, phospho-null (serine to alanine) or phospho-mimetic (serine to aspartic acid) substitutions, was differentially regulated. We show that the S73D mutant of pSREBP-1c, that mimicked a state of constitutive phosphorylation, dissociated from the SREBP-1c–SCAP complex more readily and underwent GSK-3-dependent proteasomal degradation via SCF^Fbw7^ ubiquitin ligase pathway. Pharmacologic inhibition of GSK-3 or knockdown of GSK-3 by siRNA prevented accelerated degradation of SREBP-1c. As demonstrated by MS, SREBP-1c was phosphorylated *in vitro* by GSK-3β at serine 73. Phosphorylation of serine 73 also occurs in the intact liver. We propose that GSK-3-mediated phosphorylation of serine 73 in the rat SREBP-1c and its concomitant destabilization represents a novel mechanism involved in the inhibition of *de novo* lipid synthesis in the liver.

## Abstract (precis)

We have identified Serine 73 as a novel GSK-3β site on SREBP-1c that alters its affinity for SCAP, and proteasomal degradation. Phosphorylation of Serine 73 by GSK-3β during starvation (insulin-depleted stat) may lead to lower levels of SREBP-1c; conversely, de-phosphorylation of this site may be involved in stabilizing SREBP-1c by insulin (by blocking GSK-3β action). A functional role of this site needs to be corroborated *in vivo*.

## INTRODUCTION

The sterol regulatory element binding protein-1c (SREBP-1c) is a key transcription factor that regulates *de novo* lipogenesis in the liver by activating genes involved in fatty acid and triacylglycerol synthesis [1]. The SREBP-1a isoform, a product of alternative splicing of the SREBP-1 gene, activates both lipogenic and cholesterogenic genes. A third isoform, SREBP-2, controls genes related to cholesterol homoeostasis [2]. All SREBPs are synthesized as precursor proteins that are inserted into the endoplasmic reticulum (ER) where they associate with a chaperone, sterol-cleavage activating protein (SCAP) and ER retention proteins, Insig-1 and Insig-2 (insulin-induced gene) [3]. In response to insulin, the precursor SREBP (pSREBP)–SCAP complex dissociates from Insig, is transported to the Golgi via coatamer protein complex II (COPII) vesicles where regulated intra-membrane proteolysis (RIP) yields the transcriptionally active amino-terminal fragment, nuclear SREBP-1c (nSREBP-1c). The nSREBP-1c activates transcription of many genes involved in lipid metabolism [4–6] and has been implicated in the pathogenesis of dyslipidemia and hepatic steatosis [4,7].

Although SREBPs are known to undergo phosphorylation [8–10], acetylation [11], sumoylation [12], and ubiquitination [13], phosphorylation has emerged as a key modification involved in the RIP, turnover and transcriptional activity of these proteins. A number of putative phosphorylation sites on SREBP-1 have been identified, either through direct experimentation or by *in silico* analysis. Phosphosite Plus (http://www.phosphosite.org) [14] lists 15 phosphorylation sites on SREBP-1 as putative targets of protein kinase A [15], adenosine monophosphate kinase [16], glycogen synthase kinase-3 (GSK-3) [9], cyclin-dependent kinase-1 [17], salt inducible kinase and mitogen-activated protein kinase [18–22]. Five additional sites have been identified by mass spectrometry analysis [19,23,24]. The precise identities of phosphorylation sites and the putative signalling kinases regulating the transcriptional and posttranscriptional functions of SREBP-1c have only begun to be studied.

We have previously shown that insulin treatment led to a rapid phosphorylation of pSREBP-1c and its ER to Golgi transport and RIP were tightly coupled to phosphorylation [25]. With a long-term goal to define the phosphoproteome of SREBP-1c, we purified full-length rat SREBP-1c from McA-RH7777 hepatoma cells and identified serine 73 by mass spectrometry as a novel phosphorylation site. Here, through combined analysis of site-specific mutagenesis and other molecular manipulations, we demonstrate that phosphorylation of serine 73 is involved in the ubiquitination and proteasomal degradation of SREBP-1c via ubiquitin ligase complex of F-box and WD domain containing protein 7 (SCF^Fbw7^) ubiquitin ligase pathway. We discovered that replacement of serine 73 by aspartic acid (mimicking constitutive phosphorylation), either in the full-length or nuclear SREBP-1c, resulted in increased turnover of these proteins. Moreover, we show that GSK-3-mediated phosphorylation is directly involved in this mechanism. Based on these data we conclude that activation of GSK-3 during insulin deprivation (e.g., fasting) states might lead to rapid proteosomal degradation of SREBP-1c in the liver and its ability engage in *de novo* lipid synthesis.

## EXPERIMENTAL

### Reagents

Cycloheximide, actinomycin D, MG132, insulin, LiCl, SB415286 and DAPI were purchased from Sigma-Aldrich. The restriction endonucleases (NheI, XhoI, XbaI, EcoRI, KpnI and BamHI) and recombinant GSK-3β were bought from New England Biolabs. All of the primers used were synthesized from Integrated DNA Technologies. Protein A/G plus agarose was purchased from Santa Cruz. The anti-HA, anti-Myc antibodies and SignalSilence Control siRNA, SignalSilence GSK-3α/β siRNA were obtained from Cell Signaling; anti-actin was obtained from Sigma-Aldrich; anti-SCF^Fbw7^ was obtained from Abcam and anti-SREBP-1 was bought from Becton-Dickinson and Co. GenJet Plus transfection reagent was purchased from SignaGen Laboratories and Halt™ combined protease and phosphatase inhibitor cocktails were bought from Thermo Scientific. Trypsin and Lys-C enzymes for mass spectrometry and the Dual-Luciferase® Reporter (DLR™) Assay System were obtained from Promega. SCF^Fbw7^ siRNA 1 (s30664), SCF^Fbw7^ siRNA 2 (s224357), SimplyBlue and Lipofectamine RNAiMAX were purchased from Invitrogen. AdEasy XL Adenoviral Vector System was purchased from Agilent Technologies.

### Cell culture, treatments with insulin and kinase inhibitors

Rat McA-RH7777 hepatoma cells, human embryonic kidney 293 (HEK293), AD-293 cells were cultured in complete DMEM [containing high glucose (25 mM) and 10% fetal bovine serum (FBS)]. To assess the effect of insulin treatment on the expression of nascent SREBP-1c and its maturation by proteolysis, McA-RH7777 cells were transfected with pShuttle-IRES-hrGFP-HA-pSREBP-1c-Flag plasmid. Thirty-six hours after transfection, cells were sequentially incubated in serum-free DMEM with low glucose (5 mM) for 12 h, followed by incubation in serum-free DMEM with high (25 mM) glucose either lacking or containing insulin (100 nM) for additional 8 h. For inhibitor studies, McA-RH7777 cells transfected with denoted plasmids for 36 h were grown in complete DMEM +/- inhibitors (20 mM of LiCl or 50 μM of SB 415286) for 6 h.

### Plasmid, adenovirus vectors construction and transfection/infection

The full-length rat SREBP-1c, containing either 6xHis or HA tags were amplified by PCR from rat hepatocyte cDNA using HotStar HiFidelity Taq Polymerase kit (Qiagen). The sequences of the 6xHis-tag and HA-tag forward primers (F) are as follows:

(CTAGCTAGCCCACCATGGGACATCATCATCATCATCA-CGATTGCACATTTGAAGAC) and (CTAGCTAGCCCA-CCATGGGATACCCATACGATGTTCCAGATTACGCTGGTG-GTATGGATTGCACATTTG). The reverse primer (R) (CCGCTCGAGGCTGGAAGTGACAGTGGTCCC) was common in both PCR amplifications. Thermal cycle conditions: denaturation at 95°C for 5 min followed by 35 cycles of 94°C 15 s, 50°C 1 min, 68°C 4 min; last extension at 72°C for 10 min. The pShttle-IRES-hrGFP-1 plasmid and PCR products were then digested with NheI and XhoI, purified by Ultraclean 15 DNA Purification Kit (MO BIO Laboratories) and ligated with T4 ligase (New England Biolabs). The pShuttle-IRES-hrGFP-His-pSREBP-1c-Flag and pShuttle-IRES-hrGFP-HA-pSREBP-1c-Flag plasmids were then packaged into Ad-His-pSREBP-1c-Flag and Ad-HA-pSREBP-1c-Flag viruses in AD-293 cells, respectively, according to the manual of AdEasy XL Adenoviral Vector System. The adenoviruses were amplified and purified by Vector BioLabs (Eagleville, PA). The HA-pSREBP-1c-Flag insert was then sub-cloned into pEGFP-C2 from pShuttle-IRES-hrGFP-HA-pSREBP-1c-Flag using the following primers, EcoRI-HA-F: CCGGAATTCATGGGATACCCATACGATG and Flag-XbaI-R: TGCTCTAGATTATTTGTCGTCATCATCC. The pcDNA3.1-HA-pSREBP-1c-Flag WT plasmid was constructed using HA-Tag forward primer and 1c-EcoR1-Flag-R: CCGGAATTCTTATTTGTCGTCATCATCC for amplification from plasmid pEGFP-C2-HA-pSREBP-1c-Flag. Site-directed mutagenesis was performed using QuikChange Site-Directed Mutagenesis Kit from Stratagene. For S73A, the following primers were used, SREBP-1c-S73A-F: GACACCTGCACCCTTGGCCCCTCCACCATCGGCAC and SREBP-1c-S73A-R: GTGCCGATGGTGGAGGGTCCAAGGGTGCAGGTGTC. For S73D, the following primers were used, SREBP-1c-S73D-F: GACACCTGCACCCTTGGACCCTCCACCATCGGCAC and SREBP-1c-S73D-R: GTGCCGATGGTGGAGGGTCCAAGGGTGCAGGTGTC. The pcDNA3.1-Myc-SCAP was constructed by nested PCR. First round forward primers were as follows, SCAP-minus-141F: TCTCCCGTGGTTGGAGGAAACGAG and SCAP-4162R: GGTCCAAAGAGTTGCAATCCCCAG. Inner primers are as follows, SCAP-KpnI-Myc-F: GCTTGGTACCCCACCATGGGAGAGCAGAAAC TCATCTCTGAAGAGGATCTTGGTGGTATGACCCTGACTGAAAGGCTTCG and SCAP-BamHI-R: CGCGGATCCTCAGTCCAGTTTCTCCAGCACAG. The pCS2-Myc-Ubiquitin plasmid was a gift from Dr. Patrick Ryan Potts at UT Southwestern Medical Center. The pGL4-FASN-Luciferase was constructed in our lab. Transfection assays were performed using GenJet Plus transfection reagent. The Histidine tag containing SREBP-1c was expressed in McA-RH7777 cells infected with recombinant adenovirus with a multiplicity of infection (MOI)=100. Gene knockdown was performed by transfecting SignalSilence Control siRNA or SignalSilence GSK-3α/β siRNA or SCF^Fbw7^ siRNA 1 and 2 into McA-RH7777 cells using Lipofectamine RNAiMAX reagent. To determine a role of ubiquitination, McA-RH7777 cells were co-transfected with vectors expressing HA-tagged pSREBP-1c (WT, S73A, S73D) and Myc-Ubiquitin, cultured for 24 h post-transfection in complete medium followed by 6 h incubation in medium containing MG132.

### Immuno-precipitation

McA-RH7777 or HEK293 cell monolayers (grown in 10 cm diameter Petri dishes) were rinsed with ice-cold phosphate-buffered saline (PBS) and lysed with 1 ml of ice-cold 1x cell lysis buffer (50 mM Tris/HCl pH 8.0, 150 mM NaCl, 1% Triton X-100, 0.1% SDS plus protease and phosphatase inhibitors). Cell lysates were transferred to micro-centrifuge tubes, kept on ice for 30 min and centrifuged (10 min at 16,000 ***g***, 4°C). Lysate containing 1000 μg of total protein was mixed with 7.5 μg primary antibody, and the mixture was gently rocked overnight at 4°C. Protein A/G agarose beads (80 μl of 50% bead slurry) were added to the tube that was gently rocked for additional 4 h at 4°C. The contents of the tubes were pelleted by centrifugation (2,300 ***g***, 30 s at 4°C) and the pellets were washed five times, each time with 1000 μl of 1× cell lysis buffer at 4°C. The pellets were taken up in 60 μl of 1× SDS sample buffer, denatured at 100°C for 10 min and centrifuged at 16,000 ***g***. 45 μl samples were size-fractionated by SDS-PAGE.

### Mass spectrometry (MS) analysis of SREBP-1c

The Coomassie blue-stained immune-precipitated SREBP-1c, corresponding to a bona fide pSREBP-1c band identified on a parallel gel by Western blot, was excised from the gel. Gel fragments containing SREBP-1c protein were subject to proteolysis with trypsin or trypsin and Lys-C [26]. The phosphorylated peptides isolated from the gel extracts were enriched using TiO_2_ [27] and analysed by nanoflow liquid chromatography tandem mass spectrometry (LC–MS/MS) on a LTQ Velos Orbitrap (Thermo Scientific) interfaced with an EasyLC nano LC system as previously described [28,29]. Precursor and the fragment ions were analysed at 30000 and 15000 resolutions, respectively, and searched with Mascot (Matrix Science) through a Proteome Discoverer 1.4 (Thermo Scientific) against Refseq2015 database with Rattus as the species. Variable modifications allowed were deamidation of N or Q, oxidation of M and phosphorylation S, T or Y. A precursor tolerance of 20 ppm and a fragment tolerance of 0.03 Da were used. Data were filtered at a 1% false discovery rate for high peptide confidence. The phosphopeptide sequence and site localizations were confirmed by manual inspection of the corresponding MS/MS spectra [28].

For elucidation and quantification of phosphorylations sites by the iTRAQ (isobaric tags for relative and absolute quantification) method, attachment of TMT 6-plex isobaric mass tags (Thermo) and in gel proteolysis were performed in 50% triethyl ammonium bicarbonate (TEAB) instead of 20 mM ammonium bicarbonate. Extracted peptides were re-suspended in 41 μl of anhydrous acetonitrile before adding 8 μl of a TMT 6-plex isobaric mass tag in 20 μl TEAB [30]. Phosphopeptides were not enriched on TiO_2_ columns. The combined TMT labelled peptide samples were de-salted on a C18 ZipTip (Millipore), eluted stepwise in 15%, 25% and 75% acetonitrile at basic pH, and analysed by LC–MS/MS. TMT label on the peptide N-terminus and K as variable modifications were added to the database search criteria. The ratios for the phosphorylated peptides in samples were calculated from TMT reporter ions after normalization on the median ratio from all peptides detected from SREBP-1c.

### Western blot analysis

Cell extracts or purified proteins were size-fractionated by SDS-PAGE and electro-transferred on to nitrocellulose membranes. Membrane-bound proteins were probed with HA-, Myc-, FLAG- and SREBP-1-specific antibodies as we have described in detail previously [25]. The polypeptide bands on the Western blots were scanned and quantified using Quantity One software from Bio-Rad (Hercules, CA).

### *In vitro* phosphorylation of nSREBP-1a and nSREBP-1c proteins by GSK-3β

Poly-histidine tagged rat nSREBP-1a or nSREBP-1c proteins were produced in *E. coli*. One microgram of recombinant nSREBP-1a or nSREBP-1c per aliquot was taken up in 1x NEBuffer for protein kinases, that were supplemented with or without 1000 units of GSK-3β, and 200 μM ATP. Following 2 h incubation at 30°C, protein samples were size-fractionated by SDS-PAGE and stained with SimplyBlue. Gel fragments containing polypeptide bands corresponding to nSREBP-1a and nSREBP-1c were cut out and processed for MS analysis as reported before [28].

### Luciferase assay

Dual luciferase assays and quantification were performed as previously described [28]. The HEK 293 cells were plated in 48-well plates (2×10^4^ cells/well) in DMEM containing 25 mM glucose (Day one). On the second day, 250 ng pcDNA3.1 or 220 ng pcDNA3.1-HA-pSREBP-1c-Flag (WT, S73A and S73D) or pcDNA3.1-nSREBP-1c (WT, S73A and S73D) plus 30 ng of pGL4-FASN-Luciferase DNAs were transfected into the cells. On the 4th day, cells were incubated in DMEM with or without 10% FBS for the last 8 h and harvested in 200 μl of 1x passive lysis buffer; luciferase activity was assayed using the Dual-Luciferase Reporter Assay System (Promega, Madison, WI) and quantified with a fluorometer (Turner Designs).

### Quantification of gene expression by qPCR

Trizol (Invitrogen) reagent was used to extract total RNA from HEK293 cells 48 h after transfection with pcDNA3.1-nSREBP-1c (WT, S73A and S73D). After converting total RNA into cDNA, the abundance of acetyl Co-A carboxylases (*ACC1, ACC2*), steroyl Co-A desaturase 1 (*SCD1*), fatty acid synthase (*FASN*), ATP citrate lyase (*ACLY*) was determined by quantitative PCR (qPCR) using gene-specific primers and GoTaq qPCR Master Mix (Promega, Madison, WI) on an LightCycler 480 real-time PCR system (Roche). Data were analysed using the ΔΔCT threshold cycle method. The mRNA levels of genes were normalized to that of 28S ribosomal RNA and presented as relative to the mock-transfected cells. The specificity of the PCR amplification was verified by melting curve analysis of the final products and by running products on an agarose gel. The primers are as follows. 28S-F: GACCCGCTGAATTTAAGCAT; 28S-R: GCCTCGATCAGAAGGACTTG; FASN-F: TACGACTACGGCCCTCATTT; FASN-R: CCATGAAGCTCACCCAGTTATC; ACC1-F: GAGGTGGATCGGAGATTTCATAG; ACC1-R: AGGCTCCAGATGACGATAGA; ACC2-F: CCTGTCTCAGCCTCCTAGATAA; ACC2-R: CTGACCAACCTGGTGAAACT; SCD1-F: CCTGCAGAATGGAGGAGATAAG; SCD1-R: GCCTTCCTTATCCTTGTAGGTG; ACLY-F: GCTCTGCTACCTGCTGTATTT; ACLY-R: CAACATCCTAACGCCCTACAA.

### Determination of half-lives of proteins

McA-RH7777 cells were transfected with pcDNA3.1-HA-pSREBP-1c-Flag or pcDNA3.1-nSREBP-1c (WT, S73A and S73D mutants). Cycloheximide (10 μM was added to the growth medium 36 h post-transfection to block further protein synthesis, and the rate of degradation of previously synthesized protein was assessed. Cells were maintained in serum-free medium and extracts were prepared at 0, 2, 4, 6 and 8 h following cycloheximide treatment, size-fractionated by SDS-PAGE and subject to Western blot analysis with anti-HA antibodies to probe exogenously expressed pSREBP-1c and with anti-SREBP-1 antibodies to probe total pSREBP-1c- and nSREBP-1c-specific bands that were quantified by densitometry and Quantity One software. Since the amounts of WT and mutant proteins were different right from the start (0 h), Western blots were exposed for varying durations so that ALL protein bands could be captured in the linear range of absorbance. After normalization with actin, the rates of decay were plotted to calculate the half-lives of WT and mutant SREBP-1c proteins. The rates of turnover of WT pSREBP-1c, nSREBP-1c and their respective mutants were calculated using the ‘Half Life Calculator’ tool as outlined on the Website (http://www.calculator.net/half-life-calculator.html).

### Statistical analysis

All experiments were carried out at least three times and the error bars indicating standard errors of the mean (S.E.M.) were denoted. The data were analysed by Student's two tailed *t*-test and *P* values of <0.05 were considered to be statistically significant.

### Fluorescence microscopy

Intracellular distribution of exogenously expressed EGFP-tagged SREBP-1c and DAPI (nuclear localization) stains was studied by fluorescence microscopy (Olympus IX50); co-localization analysis was done using cellSense™ software. Pictures were first taken under individual channel (DAPI, excitation wavelength 350 nM, emission wavelength 470 nM; EGFP, excitation wavelength 395 nM, emission wavelength 509 nM) and then merged into one picture. ImageJ (open source) with plugins Image CorrelationJ software packages were used to establish co-occurrence and correlation. Pearson's correlation coefficient *R*^2^ was applied to assess the SREBP-1c nuclear localization.

## RESULTS

### Intra-membrane proteolysis of SREBP-1c in McA-RH7777 rat hepatoma cells is enhanced by insulin

**Figure 1:**
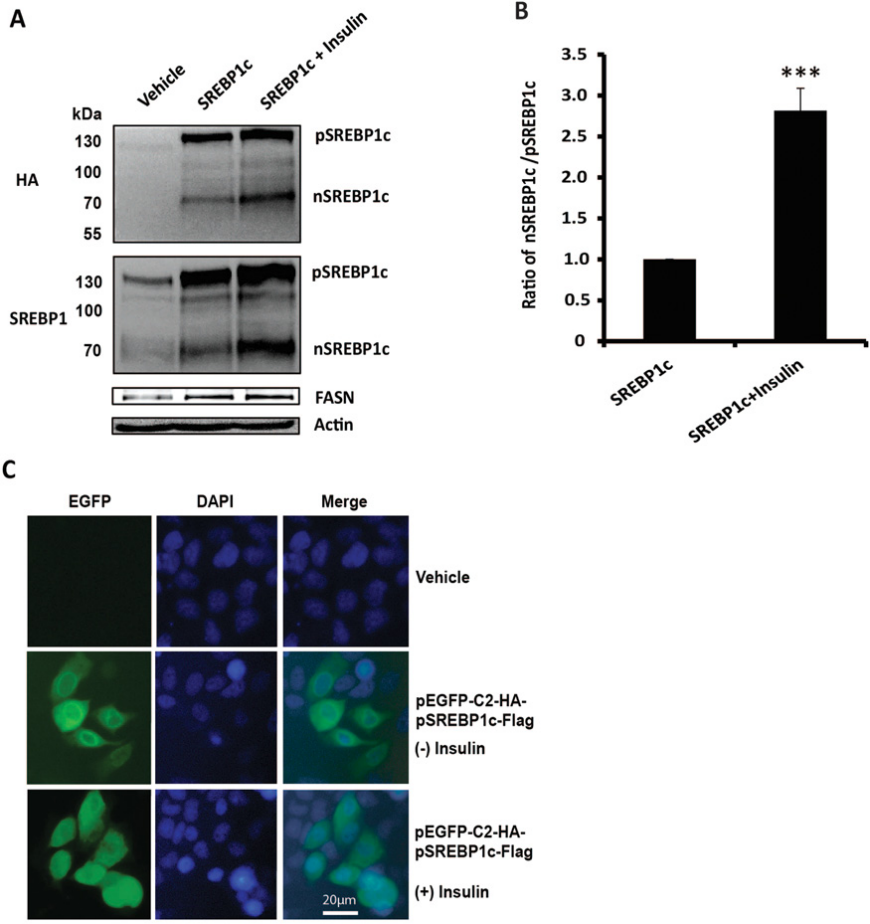
Insulin regulates the expression and intra-membrane proteolysis of the recombinant HA-pSREBP-1c-Flag protein in McA-RH7777 cells (**A**) Western blots showing the expression of HA-pSREBP-1c-Flag protein in the presence or absence of insulin. McA-RH7777 cells were transfected with pShuttle-IRES-hrGFP-HA-pSREBP-1c-Flag plasmid and 36 h after transfection cells were sequentially incubated in serum-free medium for 12 h followed by 8 h in the presence or absence of insulin and with the proteasomal inhibitor 10 μM MG132. Cell lysates were size-fractionated by SDS-PAGE, and Western blots were probed with anti-HA or anti-SREBP-1 antibodies. Cell extracts were also probed using anti-FASN and anti-actin antibodies. (**B**) Quantification of exogenously expressed HA-tagged full-length and mature SREBP-1c proteins made in vehicle- and insulin-treated cell extracts. Three independent Western blots were used to measure optical density of pSREBP1c- and nSREBP1c-specific bands. The absorbance associated with pSREBP-1c- and nSREBP1c-specific bands were normalized (by dividing these by 140 and 68, their respective molecular masses). The ratio of nSREBP-1/pSREBP-1c was set at 1.0 in vehicle-treated cells (***P value <0.001). (**C**) McA-RH7777 cells transfected with pEGFP-C2-HA-pSREBP-1c-Flag were examined under a microscope to detect green fluorescence; nuclei were stained with DAPI. Pearson's correlation coefficient *R*^2^ was applied to assess the nuclear localization of SREBP-1c. Results for the whole picture: *r*^2^=0.17 versus *r*^2^=0.48 (without versus with insulin-treated groups). Results for multiple individual cells: *r*^2^=0.50±0.02 versus *r*^2^=0.66±0.06 (without versus with insulin-treated groups) (*P*=0.015, *P*<0.05). Insulin treatment led to enhanced nuclear localization of the recombinant HA-SREBP-1c-Flag protein.

We carried out our studies of SREBP-1c phosphorylation in McA-RH7777 rat hepatoma cells that are known to recapitulate many aspects of insulin regulation of lipid metabolism [31,32]. In order to isolate sufficient quantities of protein to conduct mass spectrometry analysis of phosphopeptides, we used a strategy of over expression of recombinant pSREBP-1c in which the NH_2_ and COOH termini were specifically tagged (HA or FLAG) to facilitate detection of the full length and mature SREBP-1c with tag-specific antibodies. This strategy obviates the complications of feed-forward regulation of the endogenous SREBP-1c (that involves both RIP and transcriptional activation). To confirm physiologic regulation of exogenously expressed SREBP-1c by insulin, we transfected McA-RH7777 cells with pShuttle-IRES-hrGFP-1 to express full-length SREBP-1c with HA and Flag tags. Thirty-six hours after transfection, cells were incubated in serum-free medium for 12 h and then insulin was added for 8 h. As shown in Figure 1, both full-length and mature SREBP-1c polypeptides were robustly expressed in McA-RH7777 cells transfected with pShuttle-IRES-hrGFP-1 plasmid. As expected, HA-specific antibodies did not react with the endogenous SREBP-1c in the extracts from cells transfected with empty pShuttle-IRES-hrGFP-1 vector (Figure 1A, upper panel, left lane). In contrast, both endogenous and exogenously expressed HA-tagged pSREBP-1c and nSREBP-1c were detected by SREBP-1-specific antibody (Figure 1A, middle panel). The increased levels of nSREBP-1c detected in the Western blots probed with HA-specific antibody, indicated that the exogenously expressed pSREBP-1c underwent enhanced proteolysis in response to insulin. We quantified the levels of exogenously expressed HA-tagged pSREBP-1c and nSREBP-1c polypeptides from the Western blots of vehicle- or insulin-treated cell extracts prepared from three independent experiments. As judged by a greater ratio of nSREBP-1c/pSREBP-1c (2.81; *P*<0.001) in insulin-treated cells (Figure 1B), insulin treatment stimulated RIP of pSREBP-1c.

Since a hallmark of insulin-mediated enhanced RIP is accelerated accumulation of mature SREBP-1c in the nucleus, we assessed nuclear localization of nSREBP-1c in McA-RH7777 cells transfected with pEGFP-C2-HA-pSREBP-1c-Flag. As shown in Figure 1(C), cells were also stained with DAPI nuclear stain to assess efficiency of transfection (% of EGFP-positive compared with DAPI-positive cells). In transfected cells, co-distribution of EGFP and DAPI staining was significantly enhanced as judged by Pearson's correlation coefficient (*r*^2^=0.17 versus 0.48 in controls *versus* with insulin-treated groups, respectively). Finally, we experimentally assessed if exogenous expression of SREBP-1c in McA-RH7777 cells led to changes in the expression of downstream targets of SREBP-1c that include the gene encoding *FASN*. As shown in Figure 1(A), the steady state levels of FASN protein were significantly increased in cells overexpressing SREBP-1c. Based on these data, we suggest that tagged full-length SREBP-1c expressed in McA-RH7777 cells underwent normal ER-to-Golgi transport, intra-membrane proteolysis, nuclear localization and insulin enhanced RIP [2–4].

### Recombinant pSREBP-1c purified from McA-RH7777 cells is phosphorylated at serine 73

**Figure 2:**
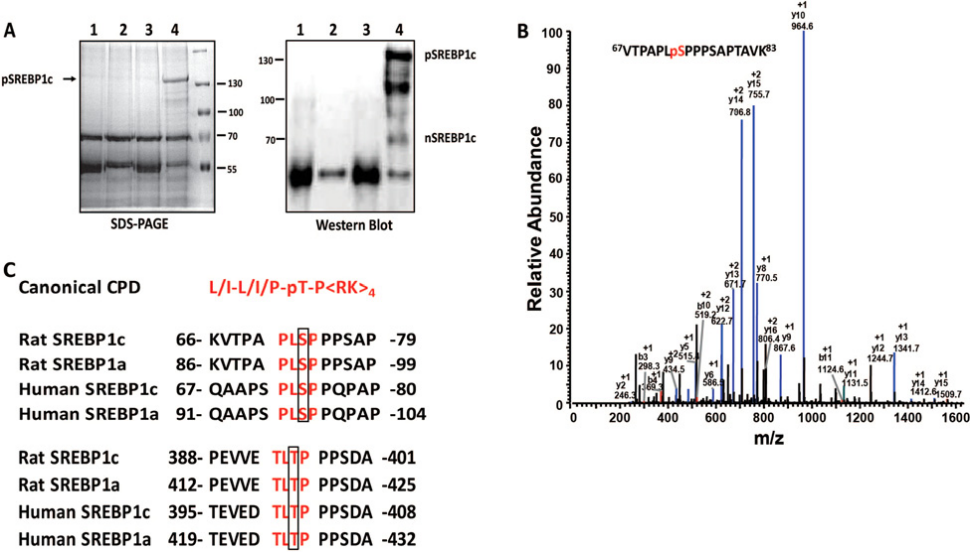
The recombinant His-tagged SREBP-1c is phosphorylated at serine 73 as characterized by LC–MS/MS (**A**) Coomassie blue-stained gel following SDS-PAGE separation of immuno-precipitated (IP) SREBP-1c and its Western blot are shown. The polyhistidine tag containing SREBP-1c was expressed in McA-RH7777 cells infected with recombinant adenovirus. Patterns of polypeptides representing cell lysates from uninfected cells IP with mouse IgG (lane 1) or anti-SREBP-1 antibody (lane 2) are shown; parallel results from extracts of recombinant adenovirus-infected cells were similarly subject to IP with mouse IgG (lane 3) or mouse anti-SREBP-1 (lane 4). (**B**) Characterization of serine 73 by LC–MS/MS. The full-length tagged SREBP-1c specific band (shown in Panel A) was excised and subjected to proteolysis and phosphopeptide enrichment. The phosphopeptides were analysed by LC–MS/MS as described in the Experimental section. The MS/MS spectrum contains a set of sequence-determining ions of the *y*-series; based on the y_8_, y_9_ and y_10_ ions the phosphorylation is located on serine 73. The sequence of the peptide encompassing serine 73 is shown (inset). (**C**) Upper panel, the amino acid sequence of the peptide containing serine 73 resembles a canonical CPD. This CPD sequence is highly conserved in the rat SREBP-1a/c and human SREBP-1a/c. Lower panel, the amino acid sequence of the peptide containing threonine 426 (human SREBP-1a) also represents a classical CPD [34].

The His-pSREBP-1c-Flag protein was concentrated by immuno-precipitation (IP) from adenovirus-infected McA-RH7777 cell extracts and size-fractionated by SDS-PAGE; on parallel lanes, proteins from whole cell extracts of uninfected and infected McA-RH7777 cells were also assessed by Western blot analysis (Figure 2A). The coomassie blue-stained band corresponding to the full-length SREBP-1c was subjected to proteolysis and analysed by LC–MS/MS. As shown in Figure 2(B), the phosphorylated serine 73 is contained in a 17-amino acid long peptide ^67^VTPAPLpSPPPSAPTAVK^83^. We discovered that the sequence surrounding serine 73 of rat SREBP-1c resembles a canonical cdc4 phosphodegron (CPD) that is a recognized substrate of the SCF^Fbw7^ ubiquitin ligase [33]. The CPD encompassing serine 73 in the rat SREBP-1c has not been previously reported; it is however evolutionarily conserved among human and rat SREBP-1c and 1a proteins and may be a putative target of GSK-3 phosphorylation (Figure 2C). Of interest Bengoechea-Alonso and co-workers [34] identified another CPD surrounding threonine 426 in human SREBP-1a. Using phospho-peptide specific antibody they further demonstrated that this threonine underwent GSK-3-dependent phosphorylation *in vitro* [35]. We should note however, that although a homologous sequence is conserved in the rat SREBP-1a and SREBP-1c (Figure 2C), our MS analysis failed to detect phosphorylation of the homologous sites by purified GSK-3β in the rat SREBP-1c and SREBP-1a (threonine 395 and threonine 419, respectively) *in vitro*.

### Site-specific mutagenesis of serine 73 reveals its role in protein stability

**Figure 3:**
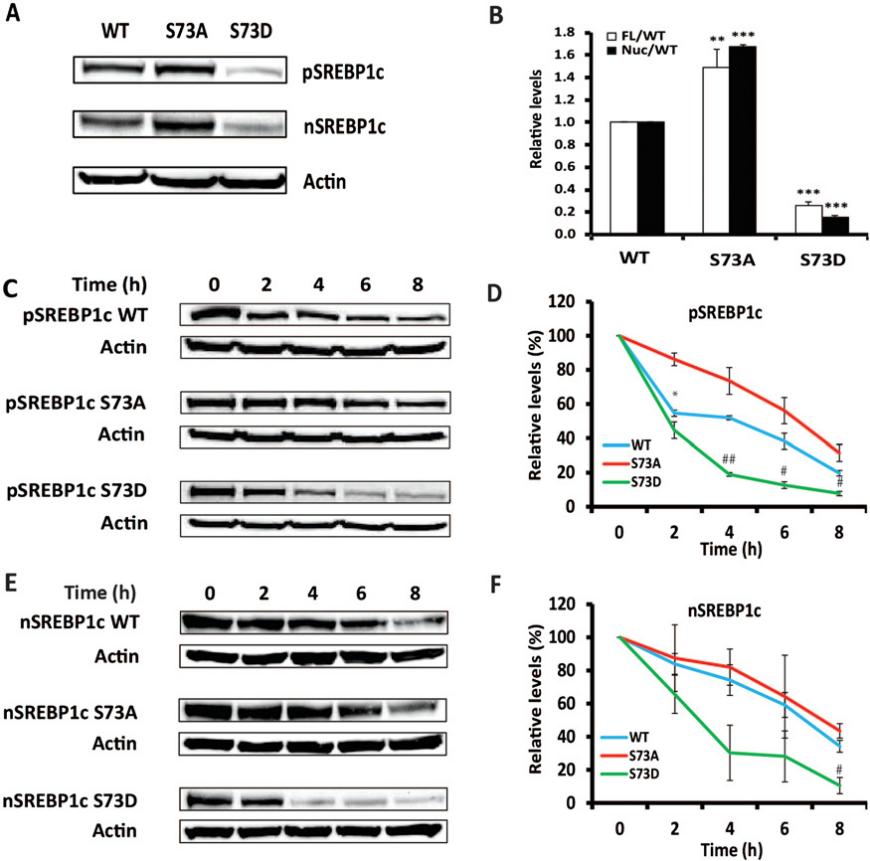
The phospho-mimetic mutation of serine 73 destabilizes the full-length and nuclear SREBP-1c (**A**) Representative Western blot showing the expression of WT pSREBP-1c and its S73A and S73D mutants in McA-RH7777 cells. McA-RH7777 cell lysates were size-fractionated by SDS-PAGE and tagged pSREBP-1c and nSREBP-1c were identified on Western blots with anti-HA antibody. Anti-actin antibody was used to assess equivalency of protein loading. (**B**) The expression of pSREBP-1c and nSREBP-1c (panel A) and another two repeats were quantified by densitometry (**P value <0.01; ***P value <0.001, as compared with WT; FL: full length; Nuc: nuclear). (**C**) and (**E**) Representative Western blots to illustrate time-dependent changes in the intracellular levels of pSREBP-1c and nSREBP-1c (WT, S73A and S73D mutants) in McA-RH7777 cells following a cycloheximide-induced block of protein synthesis. At denoted times after cycloheximide treatment cell extracts were fractionated by SDS-PAGE and Western blots were probed with anti-HA antibodies for pSREBP-1c and anti-SREBP-1 antibodies for nSREBP-1c. Exposure times were adjusted to equalize the zero time signals for WT, S73A and S73D, respectively, and rates of decay relative to zero time were plotted. (**D**) and (**F**) Quantification of Western blots from (C) and (E) and another two repeats are shown in (D) and (F), respectively (*P value <0.05, S73A compared with WT; ^#^ *P* value <0.05, S73D compared with WT; ^##^ *P* value <0.01, S73D compared with WT).

The following series of experiments were aimed at examining functional consequences of phosphorylation of serine 73 for turnover of SREBP-1c. We expressed wild type (WT) full-length SREBP-1c and its loss of function (serine to alanine; S73A) and gain of function (serine to aspartic acid; S73D) mutant counterparts in McA-RH7777 cells. The WT and mutated HA-tagged pSREBP-1c proteins were detected on Western blots using an anti-HA antibody. Gels were also probed with anti-actin antibodies to assess equivalency of protein loading. As illustrated in Figure 3, the steady state levels of pSREBP-1c protein containing the phospho-mimetic mutation (S73D) were reduced to 0.26±0.03 compared with WT pSREBP-1c and there was concomitant reduction in the levels of nSREBP-1c (0.15±0.02 of WT). In contrast, the presence of S73A mutation in the full-length SREBP-1c led to significantly higher accumulation of both pSREBP-1c (1.49±0.16; *P*<0.01) and nSREBP-1c (1.67±0.02; *P*<0.001) (Figures 3A and 3B). Based on these data we surmised that mutating serine 73 either affected the translation of the cognate mRNA encoding these polypeptides or their posttranslational stabilities. Of course, along with these two putative mechanisms, it is theoretically possible that the altered levels of SREBP-1c proteins containing S73A or S73D mutations were indirectly regulated due to unique stabilities of their cognate messenger RNAs.

To determine if reduced accumulation of the phospho-mimetic S73D mutant of SREBP-1c reflected decreased protein stability, we compared the rates of degradation of WT, S73A and S73D proteins. Since serine 73 is located in the NH_2_ terminal portion of SREBP-1c, it could influence the behaviour of both the precursor and mature forms of SREBP-1c. Therefore, we examined the stabilities of both pSREBP-1c and nSREBP-1c in McA-RH7777 cells. Twenty-four hours post-transfection, cells were incubated with or without cycloheximide (50 μM) to inhibit protein synthesis. At indicated times, cell extracts were analysed by SDS-PAGE followed by Western blots probed with anti-HA and anti-SREBP-1-specific antibodies, respectively (Figures 3C and 3E). Quantification by densitometry was used to calculate the half-lives of WT, S73A and S73D proteins (Figures 3D and 3F). The phospho-mimetic mutant (S73D) of pSREBP-1c was less stable compared with either WT or S73A mutant. The half-life of S73D mutant (2.25±0.21 h) was significantly shorter than that of S73A (4.80±0.71 h) and WT (3.25±0.07 h) for the full-length SREBP-1c protein. The half-life of the S73A pSREBP-1c mutant was increased by about 48% compared with WT; conversely, the half-life of the S73D pSREBP-1c mutant was decreased about 31% compared with WT (Figures 3C and 3D).

To experimentally compare the turnover of the WT nSREBP-1c with S73A and S73D, McA-RH7777 cells expressing these proteins were treated with cycloheximide, as outlined above. Representative Western blots of such an experiment are shown in Figure 3(E). From densitometry quantification of three independent Western blots, we calculated the half-lives of nSREBP-1c to be 5.19±0.62 h (WT), 6.68±0.66 h (S73A) and 2.46±0.51 h (S73D). We discovered that the degradation of nSREBP-1c containing S73D mutant was greatly increased and its half-life was reduced by ∼2-fold compared with WT; conversely, the half-life of nSREBP-1c containing S73A was increased about 30% compared with WT (Figure 3F). These data are consistent with the hypothesis that phosphorylation of serine 73 leads to destabilization and proteolysis of not only the full-length SREBP-1c but also the mature SREBP-1c. To our knowledge, this is the first description of a conserved site that is putatively involved in phosphorylation-dependent turnover of both pSREBP-1c and nSREBP-1c.

### Phospho-mimetic mutation of serine 73 targets SREBP-1c for proteasomal degradation

**Figure 4:**
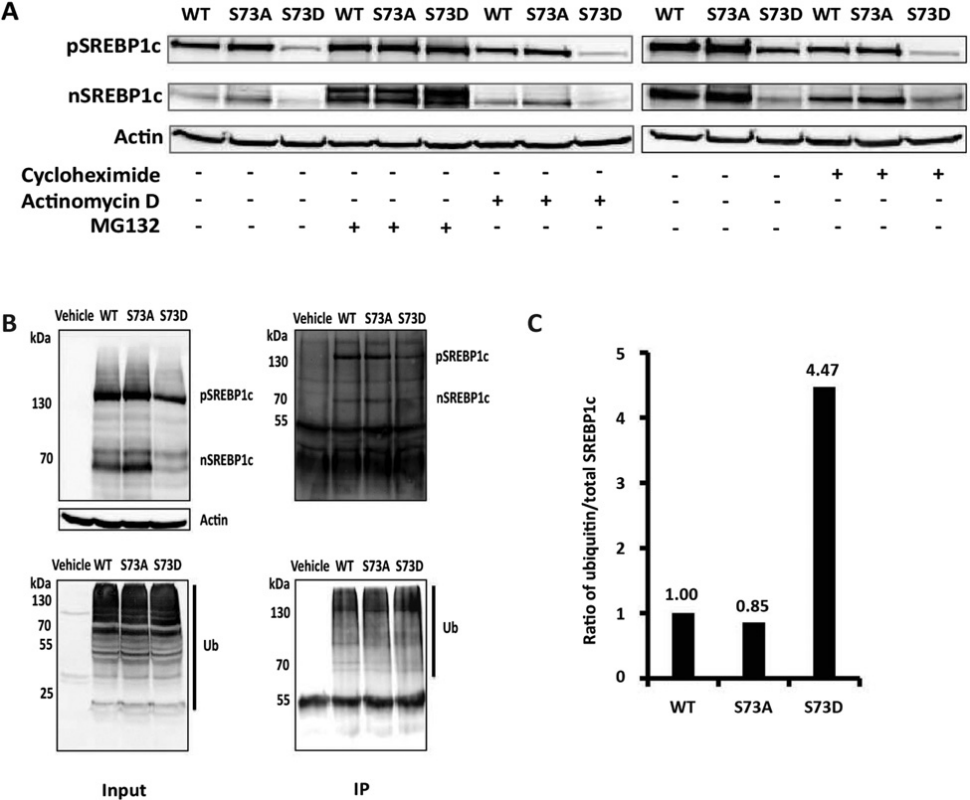
Phosphorylation of serine 73 targets SREBP-1c for ubiquitination and proteasomal degradation (**A**) Western blots showing the expression of HA-tagged full-length and mature SREBP-1c (WT, S73A or S73D mutants) in the presence or absence of a blockade of protein (cycloheximide) or RNA (actinomycin D) synthesis or proteasomal degradation (MG132). McA-RH7777 cells transfected with pcDNA3.1-HA-pSREBP-1c-Flag were either left untreated or treated with MG132 (10 μM) to inhibit proteasomal degradation or actinomycin D (50 nM), and processed for Western blot analysis as outlined in the Experimental section. Parallel cultures of McA-RH7777 cells expressing WT or mutated SREBP-1c were also incubated in medium containing cycloheximide (10 μM). Blots were probed with anti-HA or anti-actin antibodies to determine the levels of recombinant SREBP-1c or a housekeeping protein, respectively. (**B**) Blots showing interaction between SREBP-1c and ubiquitin. The McA-RH7777 cells were co-transfected with pCS2-Myc-ubiquitin and pcDNA3.1-HA-pSREBP-1c-Flag (WT, S73A or S73D); transfection with pcDNA3.1 served as a vehicle control. Cells lysates were subjected to immuno-precipitation with anti-SREBP-1 antibody. The amounts of input SREBP-1c and ubiquitin proteins in the cell extracts were determined by Western blots with anti-HA (Upper left panel) and anti-Myc (Lower left panel) antibodies. To assess interaction between SREBP-1c and ubiquitin, the IP proteins were transferred to membranes and Western blots were probed with anti-HA (Upper right panel) and anti-Myc (Lower right panel) antibodies. (**C**) Quantification of WT, S73A and S73D SREBP-1c and ubiquitin from the Western blots of immune-precipitated proteins (B, right panels) shows that S73D mutant of SREBP-1c elicited more than 4-fold greater association with ubiquitin. The results are expressed as ratios derived from arbitrary densitometry units of ubiquitin and total SREBP-1c (pSREBP + nSREBP-1c specific bands) in the corresponding lanes of two Western blots. The ratio of ubiquitin/total WT SREBP-1c was set at 1.

To experimentally test if the differential turnover of WT and mutated SREBP-1c was regulated at the mRNA or protein levels, we transfected McA-RH7777 cells with vectors driving the expression of WT or mutated pSREBP-1c and 48 h after transfection, incubated these cells for 6 h in DMEM containing cycloheximide, actinomycin D or proteasomal inhibitor MG132. Cell lysates were then analysed by SDS-PAGE followed by Western blots to assess the levels of nascent and mature forms of SREBP-1c. As shown in Figure 4(A), we observed that the differences in the steady state levels of WT, S73A and S73D were maintained in the presence of either actinomycin D or cycloheximide suggesting that mechanisms determining their differential degradation downstream of mRNA biogenesis and protein translation. In contrast, there were no apparent differences in the steady state levels of WT, S73A and S73D mutants in cells treated with MG132; even more significantly, the steady state levels of both pSREBP-1c and nSREBP-1c were similarly affected in McA-RH7777 cells treated with MG132 (Figure 4A). We should note here that increased levels of pSREBP-1c and nSREBP-1c in MG132-treated cells could be ascribed to increased expression of tagged proteins driven by the CMV promoter [36]. However, since WT and the two mutant versions of SREBP-1c are all being transcribed from the CMV promoter, their differential accumulation cannot be explained by mechanisms of differential transcription induced by CMV promoter. Therefore, the most straightforward interpretation of these data is that WT and mutated SREBP-1c proteins elicited differential stabilities, following similar rates of transcription of their cognate mRNAs that were also translated at similar rates.

The serine 73 is contained within a canonical CPD. Therefore, it is most probably involved in targeting SREBP-1c for ubiquitination followed by proteasomal degradation. We assessed this possibility experimentally and compared abilities of WT pSREBP-1c and its S73A and S73D mutants to undergo ubiquitination. The McA-RH7777 cells were co-transfected with plasmids to express HA-tagged pSREBP-1c and Myc-tagged ubiquitin, and cultured for 24 h post-transfection. Cell lysates were immuno-precipitated and subjected to Western blot analysis with anti-Myc or anti-HA antibodies, to detect ubiquitin and SREBP-1c, respectively. As expected, both the precursor and mature forms of SREBP-1c containing S73D mutation were decreased under these conditions (Figure 4B). Furthermore, ubiquitination was readily detected in WT and mutated SREBP-1c by Myc-specific antibodies (Figure 4B). Quantification of the immuno-precipitation data was done by densitometry; the ratio of arbitrary units of ubiquitin to total SREBP-1c (pSREBP-1c plus nSREBP-1c) showed that the S73D mutant of SREBP-1c was nearly 4-fold more heavily ubiquitinated as compared with the WT and S73A mutant (Figures 4B and 4C). However, the identity of the enzyme involved in phosphorylation-dependent ubiquitination remains unknown.

### Differential stabilities of WT and S73A mutant SREBP-1c are SCF^Fbw7^ dependent

**Figure 5:**
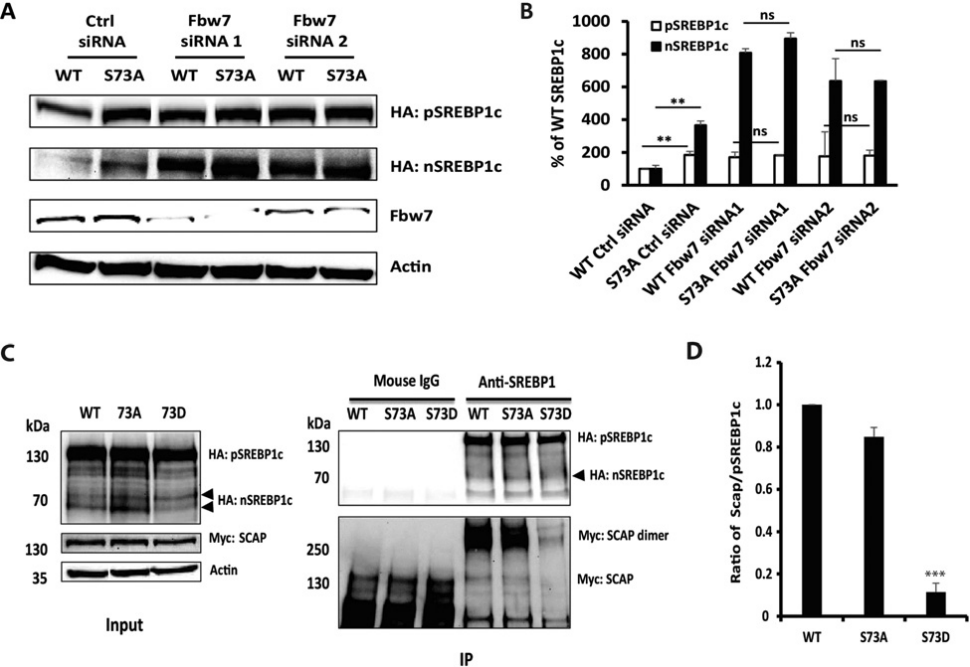
Differential stabilities of WT and S73A SREBP-1c are SCF^Fbw7^ dependent and the WT pSREBP-1c and its S73A/S73D-containing congeners differentially interact with SCAP (**A**) Western blot comparing steady state levels of full-length and mature WT or S73A mutant of SREBP-1c under conditions of knockdown of SCF^Fbw7^ by siRNA. (**B**) Quantification of pSREBP-1c and nSREBP-1c from Western blots (A and another two repeats) are shown (**P value <0.01; ns: no statistical differences, compared as labelled). (**C**) Differential association of WT pSREBP-1c and its S73A/S73D congeners with SCAP as seen in the Western blots of immuno-precipitated proteins. HEK293 cells were co-transfected with pcDNA3.1-HA-pSREBP-1c-Flag and pcDNA3.1-Myc-SCAP (10:2) and cultured in the presence of MG132 (10 μM). Total lysates were sequentially subject to immuno-precipitation with anti-SREBP-1 antibody followed by Western blot analysis using anti-HA and anti-Myc antibodies as outlined in the Experimental section. The amount of exogenously expressed SREBP-1c, SCAP and loading equivalency (actin) were assessed (left panel); specifically bound SCAP proteins in the immuno-precipitates (right panel) are also shown. (**D**) Quantification of SCAP associated with WT, S73A and S73D SREBP-1c (C, right panel) by densitometry is shown (***P value <0.001, as compared with WT).

GSK-3β has previously been shown to phosphorylate DNA-bound human nSREBP-1a (at threonine 426, serine 430 and threonine 434) and promote its degradation via SCF^Fbw7^-mediated proteasomal pathway [9,35]. Since the amino acid sequence surrounding serine 73 of SREBP-1c resembles a canonical cdc4 phosphodegradon (CPD), we tested if SCF^Fbw7^ is responsible for differential ubiquitination and stabilities of WT and S73A (non-phosphorylatable mutant). McA-RH7777 cells were transfected with HA-tagged pSREBP-1c and the expression of the nascent and mature forms of SREBP-1c were quantified in the presence or absence of SCF^Fbw7^ knockdown by two different siRNAs. As shown in Figures 5(A) and 5(B), substitution of non-phosphorylatable alanine for serine at position 73 significantly stabilized pSREBP-1c (100% versus 184.7±20.4%; *P*<0.01) and nSREBP-1c (100% versus 365.0±35.6%; *P*<0.01) proteins in cells transfected with scrambled siRNA. However, in cells where expression of SCF^Fbw7^ was knocked down using two specific siRNAs, differences in the turnover of WT and S73A SREBP-1c were eliminated. These findings clearly implicate the E3-ligase SCF^Fbw7^ in mediating degradation of the phospho-mimetic mutant (S73D) of SREBP-1c. Significantly, ubiquitination of SREBP-1c by SCF^Fbw7^ E3 ligase has been previously observed [9]. These results show that the differential stabilities of WT and S73A SREBP-1c are predicated on the phosphorylation of serine 73 that precedes their differential ubiquitination and degradation via SCF^Fbw7^. We should note however that the additional increase in S73A-containing nSREBP-1c after knockdown of SCF^Fbw7^ with siRNA (Figure 5A; column 2 versus columns 4 and 6) indicates that other phosphorylation sites may also be involved in this process.

### The WT pSREBP-1c and its S73A- and S73D-containing congeners differentially interact with SCAP

Brown, Goldstein and their colleagues have elegantly described the mechanisms by which membrane-bound precursors of SREBPs are rapidly degraded in the absence of SCAP [37]. Therefore, it is conceivable that substituting serine 73 with either alanine or aspartic acid leads to an altered conformation that changes its affinity for SCAP in the ER. To investigate this mechanism, we conducted Co-IP experiments in HEK293 cells that were transfected with pcDNA3.1-HA-pSREBP-1c-Flag (WT, S73A and S73D) and pcDNA3.1-Myc-SCAP. Total cell extracts were immune-precipitated, fractionated by SDS-PAGE and subject to Western blot analysis using anti-HA (to detect pSREBP-1c and nSREBP-1c), anti-Myc (to detect SCAP) and anti-actin antibodies. As previously described by others, we observed two bands of SCAP with the major band representing SCAP dimer [38]. The data shown in Figures 5(C) and 5(D) are consistent with the interpretation that the interactions of S73D pSREBP-1c with SCAP are about 10-fold lower than those elicited by WT pSREBP-1c (0.11±0.04 versus 1.00±0.00; *P*<0.001). Based on these data, we concluded that constitutive phosphorylation at serine 73 (as is the case for the S73D mutant of SREBP-1c) resulted in reduced affinity of the pSREBP–SCAP complex in the ER. As a result, the S73D pSREBP-1c becomes free and was targeted for rapid proteasomal degradation.

### Differential stabilities of WT, S73A-containing mutants of SREBP-1c are dependent on the activity of GSK-3

**Figure 6:**
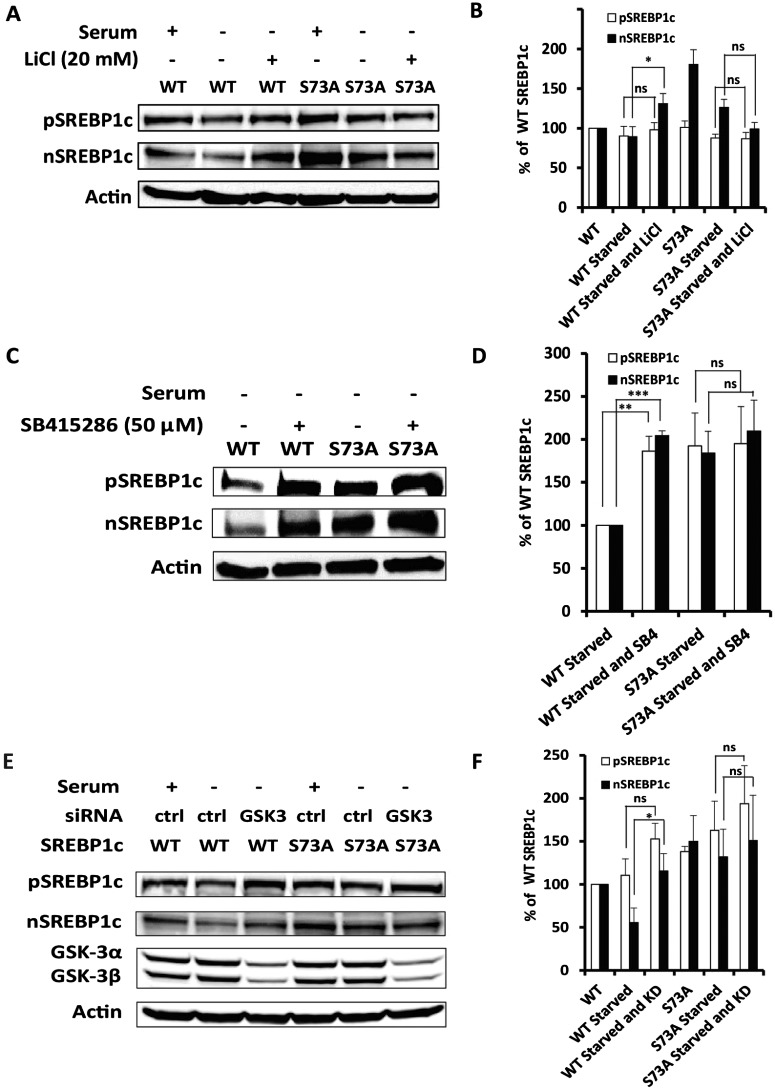
Inhibition of GSK-3 differentially regulates the turnover of WT and S73A SREBP-1c proteins (**A**) McA-RH7777 cells expressing either WT or S73A were grown in serum-depleted or complete DMEM for 24 h, followed by incubation in growth media with or without a supplement of 20 mM LiCl for 6 h. Cell extracts were subject to Western blot analysis to determine levels of pSREBP-1c and nSREBP-1c. (**B**) Quantification of nuclear and precursor SREBP-1c proteins was done from three independent Western blots (*P value <0.05; ns: not statistically significant). (**C**) McA-RH7777 cells expressing WT or S73A mutants were grown in serum-depleted DMEM +/- SB415286 (50 μM) for 24 h. A representative Western blot (C) and another two repeats were used to quantify levels of precursor and nuclear SREBP-1c shown in (D) (**P value <0.01; ***P value <0.001; ns: not statistically different). (**E**) Western blot comparing steady state levels of full-length and mature WT or S73A mutant of SREBP-1c under conditions of serum starvation and/or knockdown (KD) of GSK-3 by siRNA. (**F**) Densitometry quantification to assess the levels of the nascent and mature SREBP-1c from Western blots (E and another two repeats) under various conditions are shown (*P value <0.05; ns: no statistical differences, compared as labelled).

It is well known that insulin-induced phosphorylation of GSK3 leads to its inactivation [39]. Furthermore, the phosphorylation of serine 73 was first documented in the liver of fasted rats. The serine 73 of SREBP-1c is contained within an amino acid sequence motif predicted to be a substrate for GSK-3β-mediated phosphorylation [40]. GSK-3 has been previously implicated in phosphorylation-dependent degradation of SREBP-1a [9]. Furthermore, GSK-3 is inactivated by insulin and is active in the absence of insulin as in fasting [39]. Therefore, we experimentally assessed if GSK-3-mediated phosphorylation of Serine 73 is involved in the turnover of SREBP-1c. McA-RH7777 cells expressing either WT or S73A mutant were grown in serum-free DMEM; these conditions mimic nutritional starvation (fasting) *in vivo* that is associated with activation of GSK-3β [41]. Parallel analyses were also carried out in cells treated with LiCl that is known to potently inhibit the enzymatic activity of GSK-3 [42,43]. As shown in Figure 6, the levels of pSREBP-1c and nSREBP-1c were decreased only under conditions of depletion of serum and insulin (modelling a state of starvation and activation of GSK-3). In contrast, inactivation of GSK-3 enzyme by LiCl led to increased accumulation of WT nSREBP-1c (47.2%; *P*<0.05) but not of S73A mutant of SREBP-1c (Figures 6A and 6B).

To further corroborate the LiCl experiment suggesting a putative role of GSK-3-mediated phosphorylation in SREBP-1c turnover, we used two additional experimental strategies. First, McA-RH7777 cells expressing either WT or S73A mutant were treated with a second specific inhibitor of GSK-3 (SB415286) followed by assessing the stability of SREBP-1c. As shown in Figures 6(C) and 6(D), SB415286 treatment significantly enhanced the stability of WT; in contrast, the turnover of the S73A mutant occurred similarly in McA-RH7777 cells regardless of whether they were incubated in the presence or absence of SB415286. Secondly, we compared relative stabilities of WT SREBP-1c and its S73A-bearing counterpart in serum-starved cells, with or without siRNA-induced knockdown of GSK-3 expression. The WT nSREBP-1c levels were increased more than 2-fold (*P*<0.05) in McA-RH7777 cells in which GSK-3α/β expression was knocked down by siRNA; in contrast, the stability of S73A mutant was not significantly altered by reduced expression of GSK-3 (Figures 6E and 6F). In accordance with the prediction, the steady state levels and stability of the S73D mutant (extremely low in cells incubated in serum-starved DMEM) remained unaffected by LiCl treatment or knockdown of GSK-3 (data not shown). Based on these data we concluded that serine 73 of SREBP-1c was likely to be a target of GSK-3-mediated phosphorylation.

### GSK-3β phosphorylates nSREBP1c at serine 73 *in vitro*

**Figure 7:**
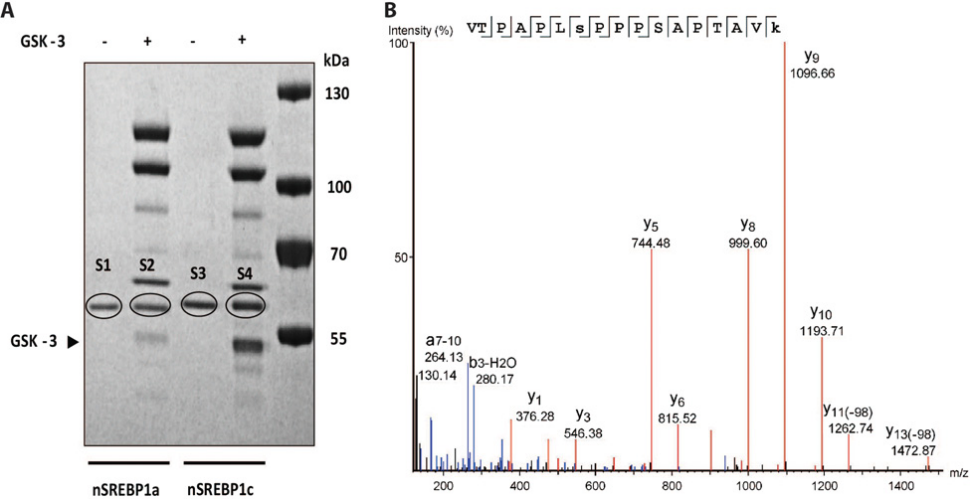
GSK-3 phosphorylates nSREBP-1c and nSREBP-1a proteins *in vitro* (**A**) The recombinant nSREBP-1a or nSREBP-1c proteins purified from *E. coli* were incubated with or without GSK-3β, size-fractionated by SDS-PAGE and stained with SimplyBlue. Lanes 1 and 3 show recombinant SREBP-1a and SREBP-1c in the absence of GSK-3, whereas lanes 2 and 4 show corresponding proteins incubated with GSK-3β. Bands marked S1, S2, S3 and S4 were cut out for MS analysis. Molecular markers are shown in the extreme right lane. (**B**) Mass spectrum demonstrating that SREBP-1c is phosphorylated at Ser73 and nSREBP-1a at Ser97. Raw data from LC–MS/MS analysis of SREBP-1c LysC/tryptic peptides was imported into PEAKS 7 and searched against the RefSeq2015 Rattus protein database confirming the PD1.4/Mascot search results for Ser73 phosphorylation of VTPAPLSPPPSAPTAVK. The spectrum has been deisotoped, converting the multi-charged ions to singly charged ions, and annotated in PEAKS. Ion y10 shows that the phosphorylation is NOT at S77 or T80. The y11-98 ion indicates that this ion has lost a phosphate and water that is diagnostic for a phosphorylation site. The b3-H_2_O shows that the phosphorylation is NOT at the T68. The ion at 264 which could be interpreted as phosphorylation on the T68, however this ion also matches the more likely internal a7-10 fragment ion which often occurs in proline-containing peptides.  Lower case ‘s’ is Ser plus phosphate (+79.97), lower case ‘k’ is Lys plus TMT 6-plex tag (+229.16).

Based on our mutational analysis, siRNA and inhibitor studies we identified Ser73 of SREBP-1c and its corresponding Ser97 of SREBP-1a as targets for GSK-3. To directly confirm that these serine residues are phosphorylated by GSK-3 we purified 6xHis-tagged rat nSREBP-1c and nSREBP-1a proteins, from *E. coli* extracts and incubated recombinant proteins with pure GSK-3β. The contents of these reactions were separated by SDS-PAGE (Figure 7A) and purified nSREBP-1a and nSREBP-1c specific protein bands (labelled S1, S2, S3 and S4) were cut out and analysed by MS (Figure 7B). The ratios for the phosphorylated peptide reporter ions (VTPAPLSPPPSAPTAVK) for GSK-3β-treated nSREBP-1a versus control (S2/S1) are as follows: median=1.20, mean=1.61, S.D.=1.11, CV=69%. The ratios for GSK-3β-treated nSREBP-1c versus control phosphor-peptide reporter ions (S4/S3) are as follows: median=1.87, mean=4.55, S.D.=8.33, CV=183%. Thus, iTRAQ MS analysis showed that Ser73 of nSREBP1c and the corresponding Ser97 of nSREBP1a were phosphorylated by GSK-3. The *in vitro* phosphorylation results thus strongly corroborate the data shown in Figure 2.

### Transcriptional regulation of downstream genes is correlated with relative stabilities of WT and mutant SREBP-1c

**Figure 8:**
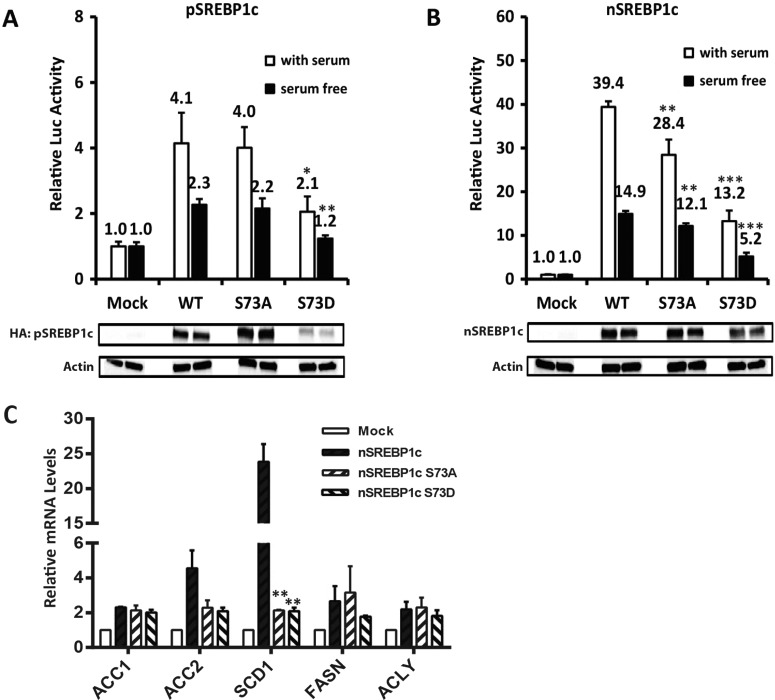
Transcriptional regulation of downstream genes is correlated with relative stabilities of WT and mutant SREBP-1c Quantification of FASN promoter-driven luciferase expression in HEK293 cells co-transfected with either pSREBP-1c (**A**) or nSREBP-1c (**B**) is shown. All experiments were repeated three times; all data were converted to fold induction ± S.E.M. (*P value <0.05; **P value <0.01; ***P value <0.001, as compared with WT under conditions with/without serum, respectively). The corresponding protein expression levels are shown below the luciferase figures. (**C**) Quantification of ACC1/2, SCD1, FASN and ACLY gene expression by qPCR is shown (**P value <0.01; as compared with WT nSREBP-1c). Relative changes in the expression of mRNAs encoding various downstream targets of SREBP-1c were done in HEK293 cells; cells were transfected with plasmids designed to express either WT nSREBP-1c or its S73A and S73D congeners and qPCR analyses were carried out as outlined in the Experimental section.

Since serine 73 is located in the NH_2_-terminal half transcriptionally active nSREBP-1c, it is conceivable that phosphorylation of this residue, in addition to being involved in degradation of SREBP-1c, may regulate its transactivation potential. One of the key downstream targets of transcriptional regulation of SREBP-1c *in vivo* is the FASN. To experimentally examine the transactivation potential of WT and mutated SREBP-1c, we co-transfected vectors expressing pSREBP-1c and nSREBP-1c (WT, S73A and S73D) individually with a FASN promoter-luciferase reporter (pGL4-FASN-Luciferase) into HEK293 cells. As illustrated in Figure 8, WT pSREBP-1c activated the pGL4-FASN-Luc promoter 4-fold and co-expression of S73A mutant of pSREBP-1c led to a similar level of transcriptional activation of the FASN promoter under normal conditions (Figure 8A). In contrast, co-transfection of the full-length pSREBP-1c containing S73D mutation was only half as effective on the FASN promoter as the WT pSREBP-1c, regardless of whether cells were cultivated with or without serum, most probably reflecting enhanced degradation of the phospho-mimetic S73D mutant protein. Our interpretation of these data is that pSREBP-1c containing S73D mutation was degraded more rapidly which was reflected by a reduced activation of the FASN gene.

Since serine 73 is located within the activation domain (consisting of NH_2_-terminal 90 amino acids) of SREBP-1c [44], we tested if this mutation affected the transactivation potential of nSREBP-1, independent of its contribution to processing of the full-length SREBP-1c. As illustrated in Figure 8(B), co-expression of the FASN promoter with WT nSREBP-1c led to nearly a 40-fold induction of luciferase activity in cells cultivated in DMEM with serum (Figure 8B). We believe that the stronger trans-acting potential elicited by nSREBP-1c reflects its immediate nuclear localization and stimulation of the FASN promoter. Compared with WT nSREBP-1c, the S73D mutant had lost about 75% of its activity on the FASN promoter, under both serum-replete and serum-free conditions (Figure 8B). Thus, constitutive phosphorylation of serine 73 was not only related to accelerated degradation of SREBP-1c but also affected its transactivation potential. Interestingly, the S73A mutation reduced the transactivation potential of nSREBP-1c to a lesser extent both in the presence (39.4 versus 28.4) or absence (14.9 versus 12.1) of serum.

We extended the promoter activation studies by directly examining the expression of five well known physiological targets of SREBP-1c in HEK293 cells. We extracted total RNA from HEK293 cells exogenously over-expressing WT or mutated SREBP-1c and analysed the downstream target mRNAs by qPCR analysis. As shown in Figure 8(C), forced expression of WT nSREBP-1c led to a 2-fold increase in the expression of acetyl co-A carboxylase 1 (ACC1), FASN and ACLY genes significantly, whereas the expression ACC2 was enhanced by 5-fold and there was 24-fold induction of SCD1. The expression of downstream target genes was lower in cells transfected with vectors designed to express S73D-containing nSREBP-1c (Figure 8C).

## DISCUSSION

SREBP-1c is a key transcription factor that regulates *de novo* lipid synthesis by activating genes involved in fatty acid and triacylglycerol biogenesis. We report here the novel discovery of phosphorylation of serine 73 in the full-length SREBP-1c produced in rat hepatoma cells. We also demonstrated that phosphorylation of SREBP-1c at serine 73 facilitates its degradation via the proteasomal pathway. The role of phosphorylation of serine 73 is somewhat controversial since its first report by Demirkan et al. [45] in the livers of rats. Based on *in silico* analysis and other theoretical considerations, these investigators assigned serine 73 as a putative ERK-1/p38 MAPK phosphorylation site. Based on theoretical analysis (GPS 2.1, http://gps.biocuckoo.org/), we also assigned serine 73 as a putative substrate of ERK-1/p38 MAPK. However, our experiments clearly revealed that the functional consequences of phosphorylation of serine 73 were not consistent with known actions of phosphorylation of SREBP-1c by MAPKs [18]. These earlier reports and our *in silico* analyses thus demonstrate the potential drawbacks of conclusions derived solely from theoretical predictions in the absence of direct experimentation.

The MAPKs are known to phosphorylate the human SREBP-1a on serine 63, serine 117 and threonine 426 (corresponding to serine 39, serine 92 and threonine 395 of rat SREBP-1c) under conditions of insulin resistance. These post-translational modifications invariably led to enhanced trans-activating capacity of human nSREBP-1a [18]. However, it remains to be determined if any of the known MAPK sites were also involved in the RIP-mediated maturation of SREBP-1a [18]. Currently, we do not know if phosphorylation of SREBP-1c by MAPKs interacts with GSK-3-mediated phosphorylation either by altering the mechanisms of RIP or by changing the transactivation potential of SREBP-1c.

Phosphorylation of serine 73 in SREBP-1c is likely to impinge on *de novo* lipid synthesis in the liver. We propose that in the fasting state induction of GSK-3β leads to phosphorylation and accelerated degradation of SREBP-1c. GSK-3β has also been shown to phosphorylate the human nSREBP-1a at threonine 426, serine 430 and threonine 434 [35]. These three sites are conserved in the rat SREBP-1c. It has been proposed that phosphorylation of these amino acids creates docking sites for the ubiquitin ligase SCF^Fbw7^, which catalyses the polyubiquitination of nSREBP-1a. At present, the relationship between serine 73 phosphorylation and previously reported GSK-3 sites is unclear. We were unable to confirm by MS the phosphorylation of the homologous sites in rat SREBP-1c and SREBP-1a incubated with pure GSK-3β. Regardless of these apparent discrepancies, we demonstrate here that phosphorylation of serine 73 is sufficient to result in marked degradation of both full-length and nuclear forms of SREBP-1c. Furthermore, ablation of phosphorylation by site-specific mutagenesis (S73A) was sufficient to stabilize SREBP-1c. The interpretation of these data is facilitated by our finding that serine 73 is encompassed in a canonical CPD recognized by SCF^Fbw7.^ We envision that under conditions of nutritional deprivation activated GSK-3β specifically phosphorylates SREBP-1c at serine 73. The S_73_PPPS_77_ of rat SREBP-1c represents a classic GSK-3 substrate motif. However, it is generally acknowledged that a subset of GSK-3 substrates must be ‘primed’ by phosphorylation prior to their phosphorylation by GSK-3 [46]. GSK-3β-catalysed phosphorylation of these substrates occurs at the fourth [47] or fifth [48] serine or threonine residue located toward the NH_2_ terminus from the primed site (pS/T1XXXpS/T2) [40]. According to this model, putative dual phosphorylation of serine 73 and serine 77 would create a high-affinity docking site for SCF^Fbw7^ [35]. We should note however, that at present we do not know the identity of the kinase(s) that phosphorylates serine 77 in order to prime the phosphorylation of serine 73. In this regard, a close proximity of serine 73 to a number of MAPK sites in the human SREBP-1a/1c [18] is intriguing.

**Figure 9:**
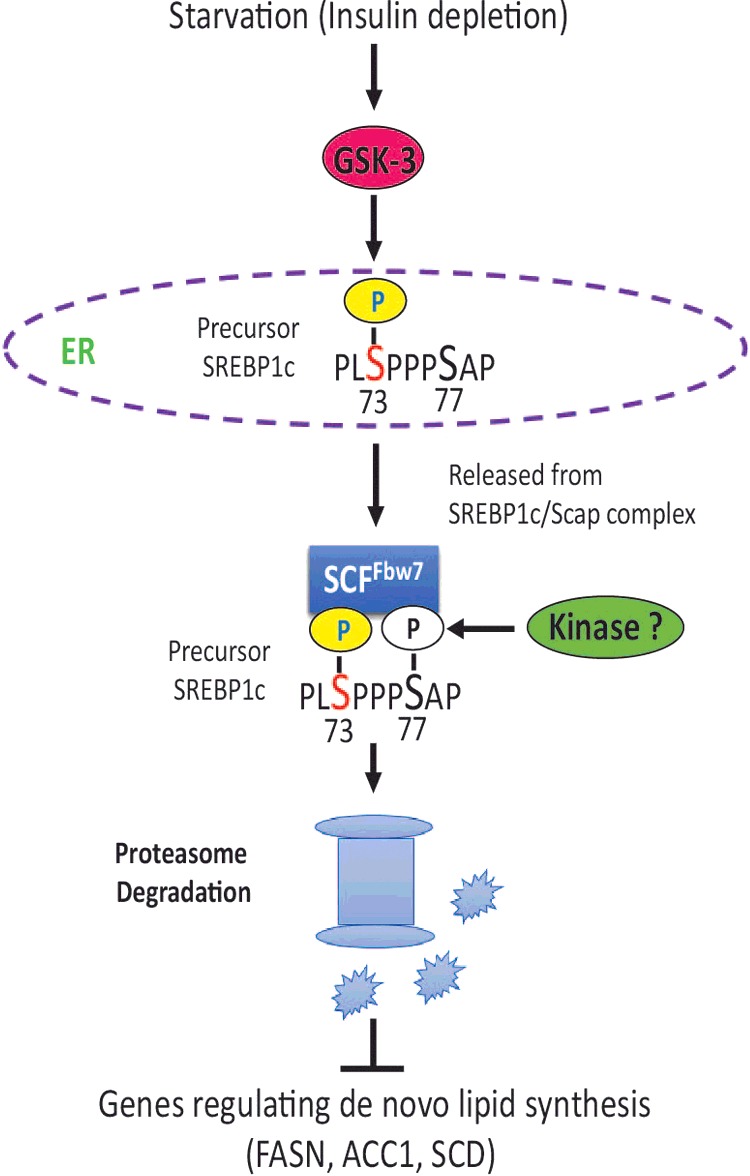
A model depicting phosphorylation-dependent proteasomal degradation of SREBP-1c Nutritional starvation or insulin deficiency leads to activation of GSK-3 that phosphorylates serine 73 of the precursor SREBP-1c in the ER. Precursor SREBP-1c, when phosphorylated at serine 73, has lower affinity for SCAP and free pSREBP-1c is targeted for SCF^Fbw7^-dependent degradation. The decreased abundance of SREBP-1c leads to reduced expression of its downstream targets involved in *de novo* lipid synthesis in the liver. The identity of the kinase predicted to phosphorylate the obligatory serine 77 in the canonical CPD recognized by SCF^Fbw7^ is currently unknown.

In addition to targeting SREBP-1c for proteasomal degradation we also observed that phosphorylation of serine 73 reduced the affinity of SREBP-1c for its ER chaperone SCAP. We therefore propose a ‘two stage’ model by which phosphorylation of SREBP-1c at serine 73 alters its 3D configuration thus facilitating its dissociation from SCAP. The free SREBP-1c is then targeted for SCF^Fwb7^-mediated ubiquitination and proteasomal degradation (Figure 9). The enzymatic activity of GSK-3 is controlled by many extracellular signals, including a potent inhibitory effect of insulin [49]. Therefore, we propose that inhibition of the phosphorylation-dependent proteasomal degradation of SREBP-1c could be an early response of insulin that potently enhances feed-forward expression of SREBP-1c and its downstream target genes. Consistent with such a mechanistic scenario, depletion of food and/or insulin would be expected to lead to activation GSK-3β and GSK-3β dependent phosphorylation of serine 73 will promote ubiquitination and proteasomal degradation of SREBP-1c.

Several metabolic proteins including 3-hydroxy-3-methylglutaryl-CoA reductase, Insigs, squalene epoxidase and CD36 are regulated by ubiquitin-proteasome system [39,50–52]. A number of transcription factors are similarly regulated by ubiquitin-proteasome pathway [53]. The F-box and WD repeat domain-containing 7 (SCF^Fbw7^) is known to target SREBPs for destruction [9]. SCF^Fbw7^ is the substrate recognition component of SCF (complex of SKP1, CUL1 and F-box protein)-type ubiquitin ligase [33] that targets cyclin E [54,55], c-Myc [56,57], Notch [58], c-Jun [59], and PGC-1α and SRC-3 [60] for degradation. SCF^Fbw7^ binds to its substrates after they have been phosphorylated within conserved motifs, called CPD. Phosphorylation within CPDs by GSK-3 facilitates binding of E3 ubiquitin ligases (e.g., SCF^Fbw7^) that catalyses ubiquitination and proteolysis [61].

It was reported earlier that the human nSREBP-1a was degraded by the ubiquitin-proteasome system following phosphorylation of threonine 426, serine 430 and serine 434 in nSREBP-1a (corresponding to threonine 395, serine 399 and serine 403 in rat SREBP-1c) [35]. Phosphorylation of these sites creates docking sites for the ubiquitin ligase SCF^Fbw7^, which catalyses the polyubiquitination of nSREBP-1a. There is emerging evidence to indicate that degradation of nSREBPs is controlled by cross-talk among multiple phosphorylated sites. Interestingly, SREBP regulates microRNAs targeting SCF^Fbw7^ [62] that in turn target mTOR for degradation [63]. Since pS_73_PPPpS_77_ of rat SREBP-1c is a classic SCF^Fbw7^ phosphodegron and based on our SCF^Fbw7^ siRNA results, we speculate that SCF^Fbw7^ is the E3 ligase involved in phosphoserine 73-dependent degradation of SREBP-1c. Apparently, the mechanisms regulating the stability of SREBP-1c have evolved to ensure that the activation of target genes is linked to the absolute levels of this key transcription factor involved in *de novo* lipid synthesis in the liver. Such a mechanism would allow the liver to respond rapidly to changes in the nutritional status and fatty acid levels and control the duration of SREBP action on its downstream targets. Consequently, a sustained activation of SREBP target genes would require a continuous supply of mature SREBP molecules through processing of precursor molecules.

In summary, using a combined strategy of unbiased mass spectrometry and functional analysis we have identified a novel GSK-3β site that was independently identified in a physiological setting *in vivo* [45] on SREBP-1c that mediates its affinity for SCAP and proteasomal degradation. Based upon our observations, this site is a prime candidate for mediating the effect of starvation to reduce SREBP-1c and conversely, de-phosphorylation of this site may mediate the effect of insulin to stabilize SREBP-1c. Establishing the role of this site *in vivo* as well as potential functional interplay with previously identified GSK-3 sites has important implications for exploring GSK-3β as a therapeutic target to treat hyperlipidaemia of type-2 diabetes and obesity [64].

## Abbreviations

ACC1: acetyl co-A carboxylase 1
ACC2: acetyl co-A carboxylase 2
ACLY: ATP citrate lyase
COPII: coatamer protein-II
CPD: cdc4 phosphodegron
ER: endoplasmic reticulum
FASN: fatty acid synthase
GSK-3: glycogen synthase kinase-3
Insig: insulin-induced gene
RIP: regulated intra-membrane proteolysis
SCD1: steroyl Co-A desaturase 1
SCF^Fbw7^: ubiquitin ligase complex of F-box and WD domain containing protein 7
SREBP: sterol regulatory element binding protein
